# Acyl-coenzyme A binding protein MoAcb1 regulates conidiation and pathogenicity in *Magnaporthe oryzae*

**DOI:** 10.3389/fmicb.2023.1179536

**Published:** 2023-04-28

**Authors:** Na Cao, Xue-Ming Zhu, Jian-Dong Bao, Li-Hong Zhu, Hao Liu, Fu-Cheng Lin, Lin Li

**Affiliations:** ^1^College of Biotechnology, Tianjin University of Science and Technology, Tianjin, China; ^2^State Key Laboratory for Managing Biotic and Chemical Treats to the Quality and Safety of Agro-Products, Institute of Plant Protection and Microbiology, Zhejiang Academy of Agricultural Sciences, Hangzhou, China

**Keywords:** *Magnaporthe oryzae*, acyl-coenzyme A binding protein, appressorium development, pathogenicity, ER-phagy

## Abstract

*Magnaporthe oryzae* is a filamentous fungus that causes rice blast. Rice blast seriously threatens the safety of food production. The normal synthesis and metabolism of fatty acids are extremely important for eukaryotes, and acyl-CoA is involved in fatty acid metabolism. Acyl-CoA binding (ACB) proteins specifically bind both medium-chain and long-chain acyl-CoA esters. However, the role of the Acb protein in plant-pathogenic fungi has not yet been investigated. Here, we identified MoAcb1, a homolog of the Acb protein in *Saccharomyces cerevisiae*. Disruption of *MoACB1* causes delayed hyphal growth, significant reduction in conidial production and delayed appressorium development, glycogen availability, and reduced pathogenicity. Using immunoblotting and chemical drug sensitivity analysis, MoAcb1 was found to be involved in endoplasmic reticulum autophagy (ER-phagy). In conclusion, our results suggested that MoAcb1 is involved in conidia germination, appressorium development, pathogenicity and autophagy processes in *M. oryzae*.

## Introduction

The filamentous ascomycete fungus *Magnaporthe oryzae*, which causes rice blast, is a model fungus used to investigate the interactions between the pathogen and the host plant (Ebbole, 2007; Dean et al., 2012). Rice blast is one of the world’s most devastating crop diseases, as it drastically reduces rice production, posing a serious threat to global food security (Wilson, 2021). Rice blast fungus has a unique infection mechanism (Martin-Urdiroz et al., 2016; Di Pietro and Talbot, 2017). Glycogen and lipids are intracellular nutrients that are continuously degraded by autophagy and transported to appressoria during the continual maturation of appressoria (Fernandez and Orth, 2018). After the turgor pressure reaches its peak, penetration pegs form and invade the plant cells, causing plant necrosis (Zhu et al., 2018; Feng et al., 2021).

Acyl-coenzyme A (acyl-CoA) binding (Acb) protein is a conserved protein in mammals, insects, fungi and plants (Rosendal et al., 1993). Acb was first discovered in mouse brain tissue as an endogenous ligand for benzodiazepine receptors because of its capacity to prevent the diazepam binding inhibitor (DBI) or endozepine (EP) from attaching to brain synaptic membranes (Chen et al., 1988). The Acb1 protein was found to have the same amino acid sequence as DBI/EP (Rose et al., 1992). The most basic function of the Acb protein is to bind to acyl-CoA and regulate other metabolic pathways (Schjerling et al., 1996). The process of acylation is necessary before fatty acids may be oxidized or produced by the organism. Long-chain acyl-CoA is a byproduct of lipid metabolism that can be employed as a signaling molecule (Roduit et al., 2004), while the Acb protein is a highly specific repository and transport intermediate. Acetyl-CoA carboxylase and mitochondrial ATP/ADP translocase have been found to be shielded by the Acb protein from being inhibited by long-chain acyl-CoA esters. Acb protein also stimulates mitochondrial acyl-CoA synthase to synthesize acyl-CoA (Rasmussen et al., 1993). In both human and mouse cells, the Acb protein regulates autophagy (Duran et al., 2010), which is inhibited by both the intracellular depletion of Acb proteins and their addition to the extracellular environment. Acb protein also regulates the basic functions of many cells (Burton et al., 2005). Gaigg et al. indicated that Acb protein affects the transport of vesicles and the biosynthesis and structure of membranes (Gaigg et al., 2001). In *Aspergillus oryzae*, the Acb protein can specifically bind medium-chain and long-chain acyl-CoA esters without binding coenzyme A, nonesterified fatty acids, acylcarnitines, and some nucleotides (Rasmussen et al., 1993). However, in pathogenic fungi, the biological function of the Acb protein has not been elucidated.

Autophagy is an evolutionarily conserved intracellular degradation process involved in maintaining intracellular homeostasis (Glick et al., 2010; Dufey et al., 2014; Cheng et al., 2018). The *MoATG1-9, MoATG12-14, MoATG16, MoATG18* of *Magnaporthe oryzae* participated in autophagy pathway, affecting the growth and development of conidia (Gao et al., 2013; Zhu et al., 2018). Atg8 protein is a ubiquitin-like protein composed of 121 amino acids with a size of 16 kDa, which is closely related to the synthesis of autophagosomes (Liu et al., 2010; Zheng et al., 2015). Since the synthesis and lipidation of Atg8 are enhanced under autophagy-induced conditions, it has been used by various studies as a reliable marker for autophagy induction (Ichimura et al., 2004; Yoshimoto et al., 2004; Nakatogawa et al., 2007). ER-phagy is a selective autophagic pathway used to remove misfolded or unfolded proteins within the ER in mammalian cells. Sec62 is a component of the transposon complex and has recently been recognized as an ER-phagy receptor during the stress recovery phase of the mammalian ER (Fumagalli et al., 2016). Studies have shown that *Arabidopsis thaliana* Sec62 is required for plant development and may act as an ER-phagy receptor in plants (Hu et al., 2020). A homolog of the *S. cerevisiae* Acb protein was identified in *M. oryzae* and named MoAcb1. We found that MoAcb1 is involved in a range of physiological activities, including appressorium formation, and pathogenicity of *M. oryzae*. Interestingly, we found that MoAcb1 negatively regulates ER-phagy, thus affecting the biological function of *M. oryzae*. Our results enrich our understanding of pathogenic fungi and provide new insights for exploring the function of *M. oryzae*.

## Results

### Identification of the Acb1 protein in *Magnaporthe oryzae*

**Figure 1:**
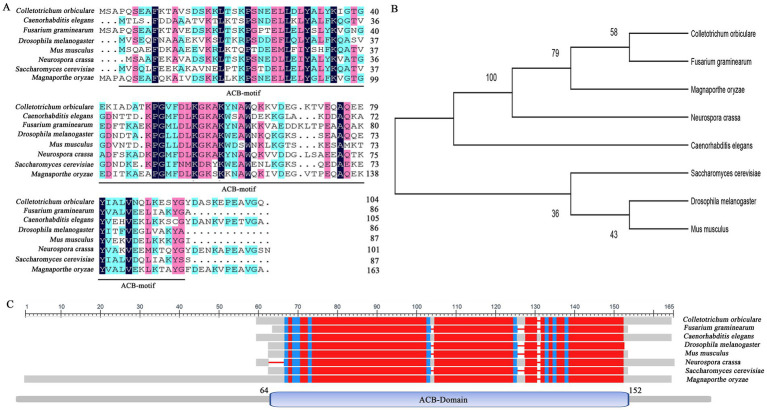
Sequence alignment of the amino acids of Acb1 in different species. **(A)** The amino acids of ACB1 domain were sequence-aligned by DNAMAN 8. Comparative sequences were obtained from *Magnaporthe oryzae* (MoAcb1), *Colletotrichum orbiculare* (TDZ22682.1), *Caenorhabditis elegans* (NP_491412.1), *Fusarium graminearum* (XP_011327983.1), *Drosophila melanogaster* (NP_523952.2), *Mus musculus* (NP_031856.1), *Neurospora crassa* (XP_962380.1), and *Saccharomyces cerevisiae* (NP_011551.3). **(B)** 500 bootstrapped ML (maximum likelihood) tree generated by MEGA v11. **(C)** The amino acids of Acb1 were sequence-aligned by SmartBLAST.

We used the amino acid sequence of *S. cerevisiae* Acb1 to search the *M. oryzae* Genome Database.^1^ We identified an Acb1 homolog (MGG_06177) that shared 43.66% amino acid similarity with ScAcb1, which we named MoAcb1. MoAcb1 showed high homology with its orthologs in other fungi, including CoAcb1 of *Colletotrichum orbiculare* (66.99% identity), CeAcb1 of *Caenorhabditis elegans* (50.59% identity), FgAcb1 of *Fusarium graminearum* (63.81% identity), DmAcb1 of *Drosophila melanogaster* (43.68% identity), MmAcb1 of *Mus musculus* (40.45% identity), and NcAcb1 of *Neurospora crassa* (66.67% identity). The MoAcb1 protein contains the ACB domain (64aa-152aa), which is also very conserved in multiple species (Figure 1). The results show that Acb1 is well conserved in various organisms.

### MoAcb1 affects conidial formation of *Magnaporthe oryzae*

**Figure 2:**
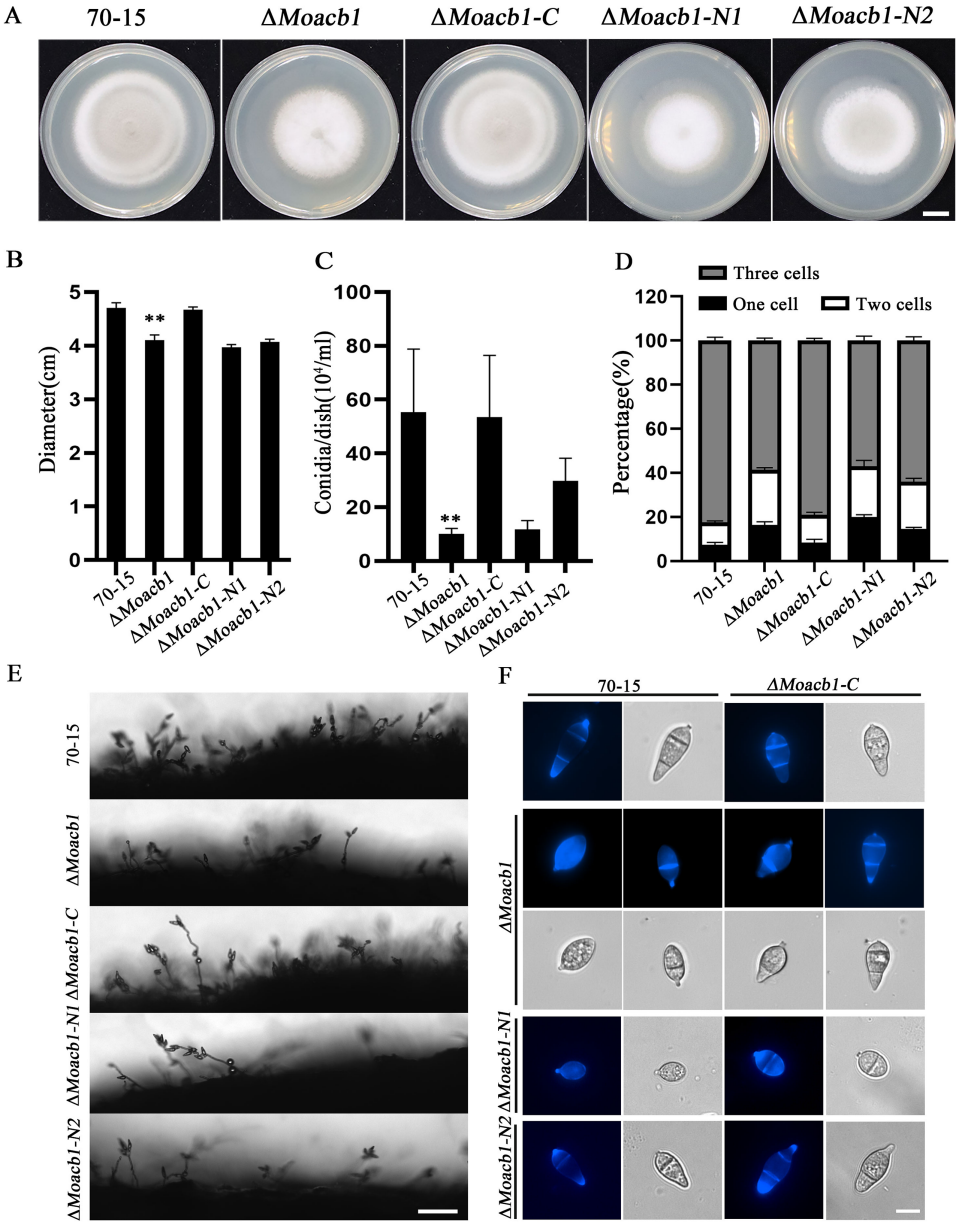
MoAcb1 is involved in conidial development of *M. oryzae*. **(A)** Conidial morphology of five strains grown in CM for 8 days. Bar, 1 cm. **(B)** Measurement and analysis of the colony diameter of five strains grown in CM for 8 days, with the standard deviation indicated by error bars. *T*-test was used to test for significant differences. **p < 0.01. **(C)** The conidial yields of five strains grown in CM for 8 days, with the standard deviation indicated by error bars. *T*-test test was used to test for significant differences. **p < 0.01. **(D)** The three different types of conidia were statistically analyzed in the five strains, with the standard deviation indicated by error bars. *T*-test was used to test for significant differences. **(E)** Observed conidiophore of the five strains under an electron microscope. Bar, 50 μm. **(F)** Conidia of the five strains were stained with calcofluor white (CFW), and the septum was stained blue. Bar, 10 μm.

To investigate the biological role of MoAcb1 in *M. oryzae*, we generated the ∆*Moacb1* mutant by homologous recombination, and then analyzed the growth and appressorium formation of different strains (Figure 2A). Compared with 70-15 and ∆*Moacb1-C*, the vegetative growth of the ∆*Moacb1* mutant was slightly slowed by approximately 20% (Figure 2B), and the production of conidia was significantly lower than that of 70-15 (Figures 2C,E). In terms of conidial morphology, the ∆*Moacb1* mutant is also significantly different from 70-15. Most conidia of the 70-15 strain have two septa, while most conidia of ∆*Moacb1* mutant have only one septum or no septum, and the conidia of ∆*Moacb1-C* return to the normal morphology (Figures 2D,F). Since the MoAcb1 protein contains an Acb binding domain, we divided its sequence into two segments, N1 (1–189 bp) and N2 (190–456 bp), and merged it into the ∆*Moacb1* mutant to obtain the ∆*Moacb1-N1* and ∆*Moacb1-N2* strains, respectively. Their growth rate, conidia morphology and conidiation are similar to those of the ∆*Moacb1* mutant (Figures 2B–F), indicating that only the full-length complement can return to normal. Taken together, these data suggest that MoAcb1 affects the growth and conidial formation of rice blast fungus.

### MoAcb1 affects appressorium formation in *Magnaporthe oryzae*

**Figure 3:**
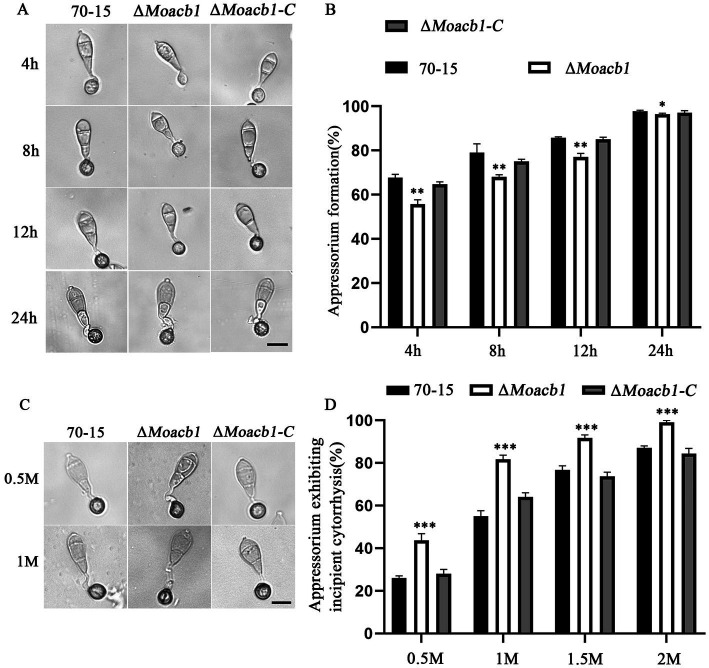
MoAcb1 is important for appressorium formation. **(A)** The conidial suspensions of the 70-15, ∆*Moacb1* and ∆*Moacb1-C* strains were inoculated on the hydrophobic surface to photograph appressorium formation at different times. Bar, 20 μm. **(B)** The formation rate of the appressorium was calculated and statistically analyzed, with the standard deviation indicated by error bars. *T*-test was used to test for significant differences. *p < 0.05, **p < 0.01. **(C)** Cell collapse of the 70-15, ∆*Moacb1* and ∆*Moacb1-C* strains in different concentrations of glycerol. Bar, 20 μm. **(D)** Cell collapse rates were calculated and statistically analyzed, with the standard deviation indicated by error bars. *T*-test test was used to test for significant differences. ***p < 0.001.

Turgor pressure accumulation and appressorium formation are key in the infection stage (Cai et al., 2022). We analyzed these processes by observing the formation of appressoria and measuring the size of the appressorium. The results showed that appressorium formation in the Δ*Moacb1* mutant was relatively delayed on the hydrophobic surface compared with that in the 70-15 and complemented strains, with a significant difference at 4 h post incubation (hpi), 8 hpi and 12 hpi but little difference at 24 hpi (Figures 3A,B). Therefore, we used 0.5–2 M glycerol for the appressorium collapse assay. By adding different concentrations of glycerol, we found that when the external glycerol concentration reached 1 M, the appressorium collapse rate of the Δ*Moacb1* mutant was significantly higher than those of 70-15 and ∆*Moacb1-C*. Only 55% of 70-15 appressoria had collapsed, while 85% of Δ*Moacb1* mutant appressoria had collapsed, indicating that the Δ*Moacb1* mutant affected turgor pressure accumulation (Figures 3C,D).

### MoAcb1 affects the pathogenicity of *Magnaporthe oryzae*

**Figure 4:**
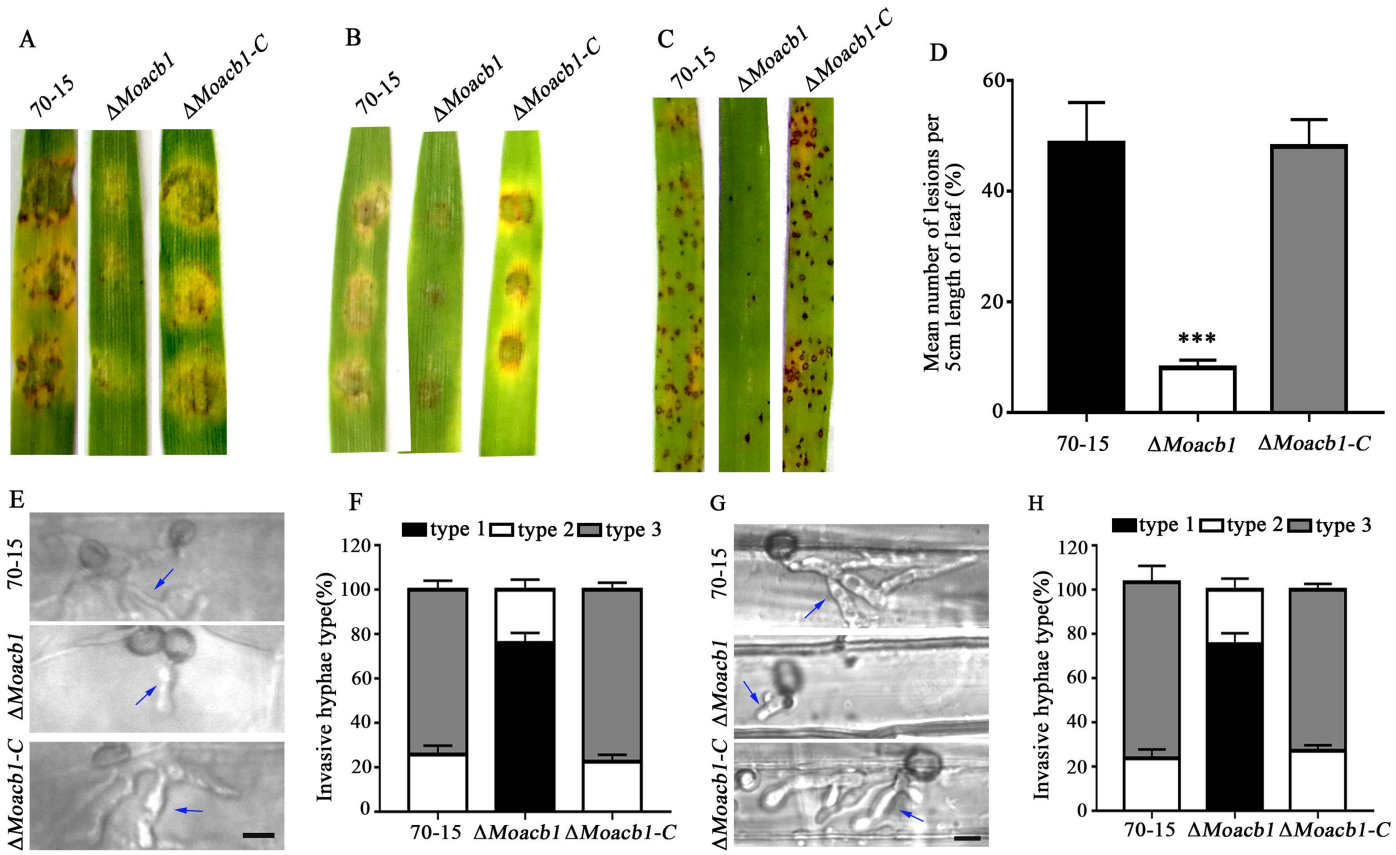
MoAcb1 affects the pathogenicity of *M. oryzae*. **(A)** Mycelial plugs of the 70-15, ∆*Moacb1* and ∆*Moacb1-C* strains were placed on isolated barley leaves, and photographs of pathogenicity were taken 4 days later. **(B)** Conidial suspensions of the 70-15, ∆*Moacb1* and ∆*Moacb1-C* strains were inoculated on isolated barley leaves, and photographs of pathogenicity were taken 4 days later. **(C)** Conidial suspensions of the 70-15, ∆*Moacb1* and ∆*Moacb1-C* strains were sprayed on isolated rice leaves, and photographs of pathogenicity were taken 5 days later. **(D)** Quantification of the lesions number per 5 cm length of rice leaf. Asterisk represents significant difference (*p* < 0.001). **(E)** Pictures were taken of the leaves infected by wild type 70-15, the ∆*Moacb1* mutant and complement strain at 36 hpi. The arrow points to the invasive hyphae. **(F,H)** Infection types produced by different strains (70-15, ∆*Moacb1* and ∆*Moacb1-C*) were counted, and the experiment was repeated three times with three replicates each time. **(G)** Pictures were taken of 70-15, ∆*Moacb1* and ∆*Moacb1-C* strains infecting leaf sheath at 36 hpi. The arrow points to the invasive hyphae.

To further explore the effect of MoAcb1 on pathogenicity, we used barley and rice leaves for pathogenicity analysis. The 70-15, ∆*Moacb1* mutant and ∆*Moacb1-C* strains were inoculated into isolated barley leaves. The results showed that the ∆*Moacb1* mutant caused only very small disease spots when compared to the severe disease spots produced by the 70-15 and complemented strains (Figure 4A). Suspensions of strain conidia (5 × 10^4^ conidia/ml) were inoculated in barley leaves after 4 days, and the ∆*Moacb1* mutant had fewer lesions than 70-15 and the complemented strains (Figure 4B). Similarly, only a few small brown spots were observed on rice leaves infected with ∆*Moacb1* mutant conidia after the conidial suspension (5 × 10^4^ conidia/ml) was sprayed onto 2-week-old susceptible rice seedlings for 7 days (Figures 4C,D). In order to further analyze the infection ability of Δ*Moacb1* mutant, we observed the infection levels of strains on leaf sheath and leaf, respectively. For the convenience of statistics, invasive hyphae are divided into three types: Type 1 has only one short invasive hyphae; Type 2 has two invasive hyphae; Type 3 forms three or more long invasive hyphae. After 36 h of infection of leaves (Figure 4E) and leaf sheaths (Figure 4G), it was found that the invasive hyphae of Δ*Moacb1* mutant was mainly type 1, while the invasive hyphae of wild-type 70-15 and the complemented strains was mainly type 3 (Figures 4F,H). The findings demonstrated the significance of MoAcb1 as a pathogenic component.

### Subcellular localization of MoAcb1 protein

**Figure 5:**
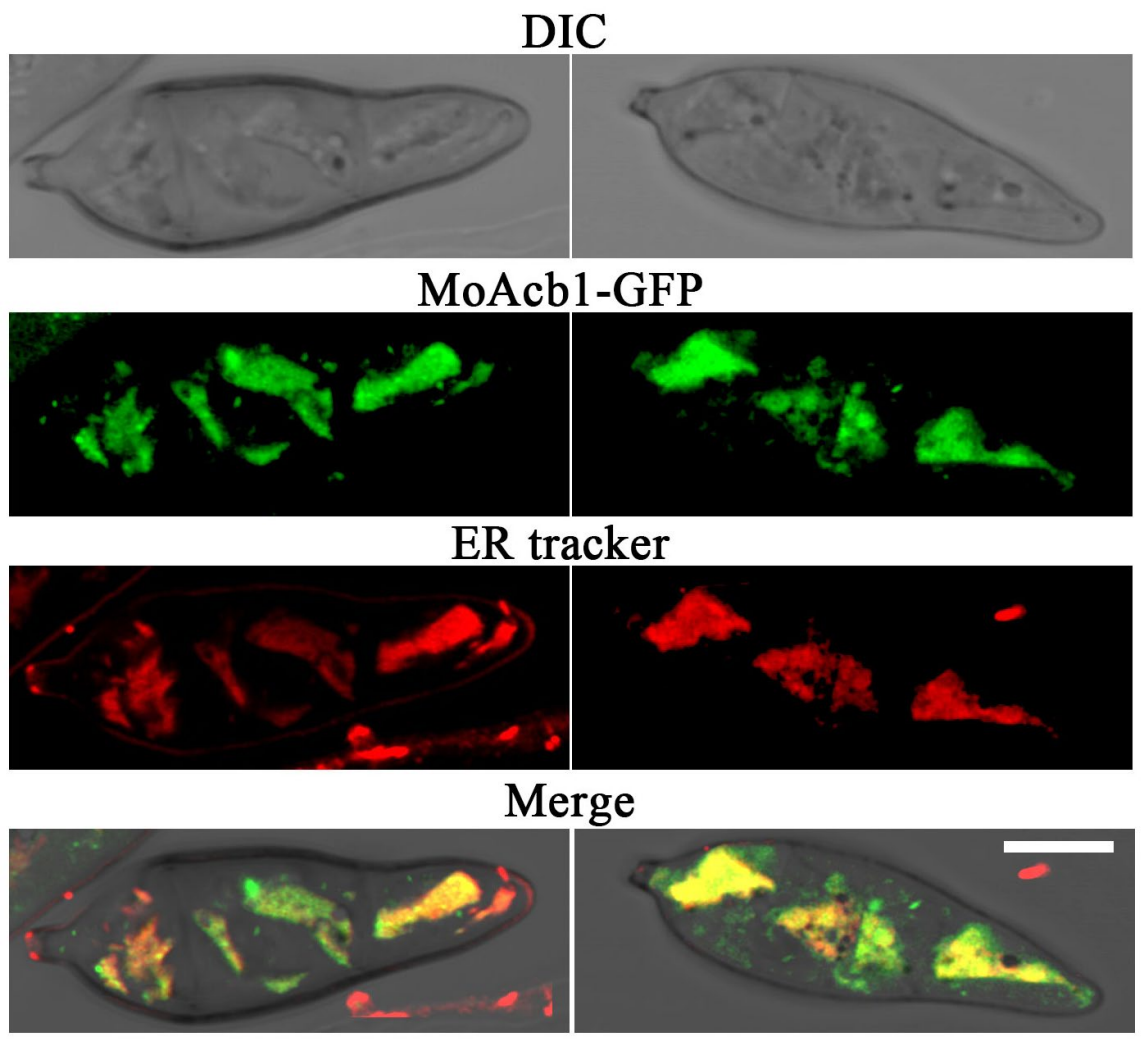
Subcellular localization of MoAcb1. Colocalization of MoAcb1-GFP and ER tracker Red in the conidia. Bar, 5 μm.

To explore the intracellular localization of the MoAcb1 protein, We observed the MoAcb1-GFP strain under a Laser confocal microscope and MoAcb1-GFP was found in the ER. Collecting conidia from complete medium (CM) and stained with ER tracker. As shown in Figure 5, we observed that the conidial cells contained multiple fluorescent spots that overlapped with the ER tracker. The experimental results demonstrated that MoAcb1 is mainly localized in the ER of *M. oryzae*.

### Deletion of *MoACB1* delays glycogen utilization and degradation

**Figure 6:**
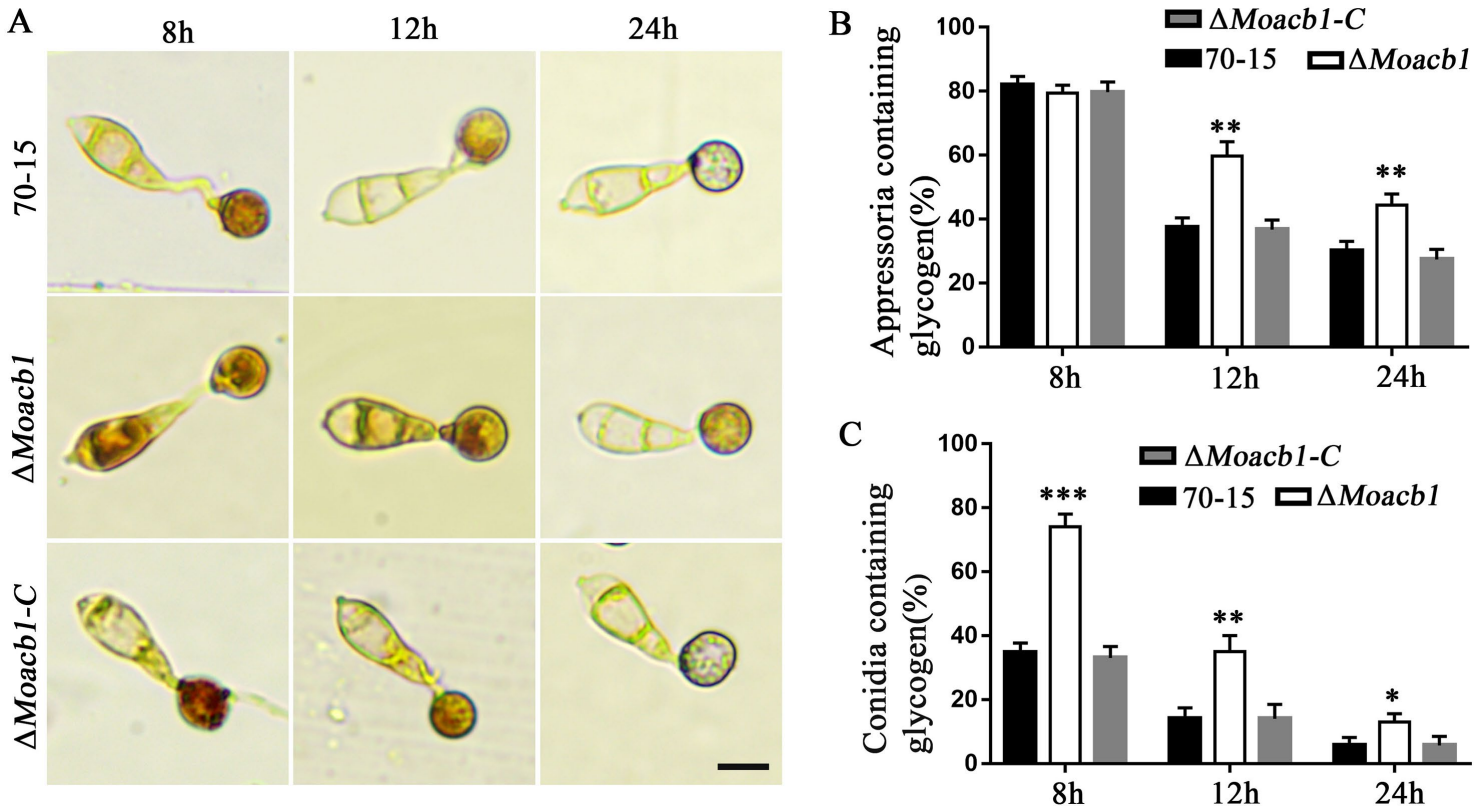
Glycogen utilization during appressorium development. **(A)** Distribution of glycogen during appressorium development. Samples were stained with KI/I2 solution at different time periods, and glycogen is dark brown under the microscope. Bar, 10 μm. **(B)** Proportion of appressoria containing glycogen, with standard deviation indicated by error bars. *T*-test was used to test for significant differences. **p < 0.01. **(C)** Proportion of conidia containing glycogen, with standard deviation indicated by error bars. *T*-test test was used to test for significant differences. *p < 0.05, **p < 0.01, ***p < 0.001.

In *M. oryzae*, to raise the turgor pressure of the appressoria and speed up penetration into the host (Thines et al., 2000), the lipids and glycogen accumulated in conidia are degraded and then transported through germ tubes to the appressoria (Howard et al., 1991; Wilson and Talbot, 2009). Due to the impaired appressorium turgor pressure of the ∆*Moacb1* mutant, we decided to study the distribution of glycogen and lipid droplets of the ∆*Moacb1* mutant during appressorium formation. Conidial germination was induced on the hydrophobic surface, and after KI/I_2_ staining, it was observed that there was abundant glycogen in the conidia, germ tubes and nascent appressorium of the 70-15 and ∆*Moacb1* mutant strains at 0 h and 4 h, but the utilization of glycogen in the conidia in the ∆*Moacb1* mutant was significantly hindered at 8 hpi (Figure 6A). In wild-type 70-15, glycogen in 65.3% of conidia is utilized, while only 26% of the conidia glycogen in the ∆*Moacb1* mutant were utilized (Figure 6C). At 24 hpi, 70.0% of the 70-15 appressoria were degraded and utilized, while 55.6% of the appressoria of the ∆*Moacb1* mutant were degraded and utilized (Figure 6B). Unlike for glycogen, the strains did not differ significantly in lipid droplet translocation. The results showed that MoAcb1 is essential for the utilization and degradation of glycogen.

### MoAcb1 affects the utilization of certain carbon and nitrogen sources by *Magnaporthe oryzae*

Since MoAcb1 is an acyl-CoA-binding protein, it specifically binds medium-chain and long-chain acyl-CoA esters and regulates lipid metabolism to some extent (Mogensen et al., 1987). Therefore, we performed experiments on the utilization of a variety of nonfermented carbon sources by the 70-15 and ∆*Moacb1* mutant. For this purpose, we replaced 1% of the glucose in the base minimal medium (MM) with other carbon sources, including sodium acetate, olive oil, palmitic acid, ferulic acid, and tetradecanoic acid (Supplementary Figure S1A). The results showed that the ∆*Moacb1* mutant had different utilization rates of the different carbon sources. The utilization rate of the carbon sources was calculated by inoculating the three strains (70-15, ∆*Moacb1* and ∆*Moacb1-C*) on MM with 1% glucose from a carbon source as the control. The results showed that the utilization rate of the ∆*Moacb1* mutant for three of the carbon sources was higher than that of 70-15, and the growth-promoting effects were 74.3% for sodium acetate, 72.2% for olive oil, 72.3% for palmitic acid. However, the utilization rate of the tetradecanoic acid by ∆*Moacb1* mutant was lower than that by 70-15 (Supplementary Figure S1B). The results indicated that the deletion of the *MoACB1* gene affects the utilization of different carbon sources.

After analyzing the carbon source utilization of the mutant, we hypothesized that the gene also affected nitrogen source utilization. The NaNO_3_ concentration of the only nitrogen source in the MM was changed to 10 mM as a control, and NaNO_2_, (NH_4_)_2_SO_4_, NH_4_NO_3_, glutamine (Gln) and histidine (His) were selected as the test nitrogen sources (Supplementary Figure S1C). The data were analyzed, and it was found that the utilization rate by the Δ*Moacb1* mutant of (NH_4_)_2_SO_4_, NH_4_NO_3_ and His was higher than that by the 70-15 strain, showing a growth-promoting effect. The utilization rate of NaNO_2_ and Gln by the Δ*Moacb1* mutant was lower than that by the 70-15 strain, showing a growth-inhibiting effect (Supplementary Figure S1D). These results indicated that the deletion of the *MoACB1* gene also affects the utilization of different nitrogen sources.

### MoAcb1 is involved in the regulation of hypertonic stress responses and DNA replication stress responses

The key of *M. oryzae* infection is its ability to resist external environmental stresses (Li et al., 2012). To detect strain sensitivity to hypertonic stress, 70-15, Δ*Moacb1* mutant and ∆*Moacb1-C* were inoculated on CM plates containing 0.5 M potassium chloride, 1 M sucrose, 0.5 M sodium chloride, and 1 M sorbitol (Supplementary Figure S2A). Using CM as a control, the inhibition rate was calculated, and the experimental data were calculated from the results of three independent experiments. As shown in Supplementary Figure S2C, the loss of the *MoACB1* gene resulted in increased resistance to ionic hyperosmotic stress and enhanced growth fitness, indicating that the *MoACB1* gene plays an important role in adaptation to hyperosmotic stress.

The completion of DNA replication controls the initial development of appressorium and the appearance of penetration pegs (Saunders et al., 2010; Oses-Ruiz et al., 2017). Hydroxyurea (HU) and methyl mesylate (MMS) are DNA damage agents. To elucidate the cellular function of MoAcb1 in *M. oryzae*, we investigated the sensitivity of the ∆*Moacb1* mutant to HU and MMS. ∆*Moacb1* mutant and ∆*Moacb1-C* and 70-15 were inoculated on CM plates containing 20 mM HU and 0.02% MMS. The inhibition rate was calculated by using CM as a control. Loss of the *MoACB1* gene increased strain resistance to DNA-damaging agents and enhanced growth fitness. In particular, the growth rate of the ∆*Moacb1* mutant inoculated on HU was significantly higher than that of strain 70-15, which was more responsive to DNA replication pressure, indicating that *MoACB1* played a role in the DNA replication stress response (Supplementary Figures S2B,D).

### MoAcb1 is involved in the regulation of ER-phagy

**Figure 7:**
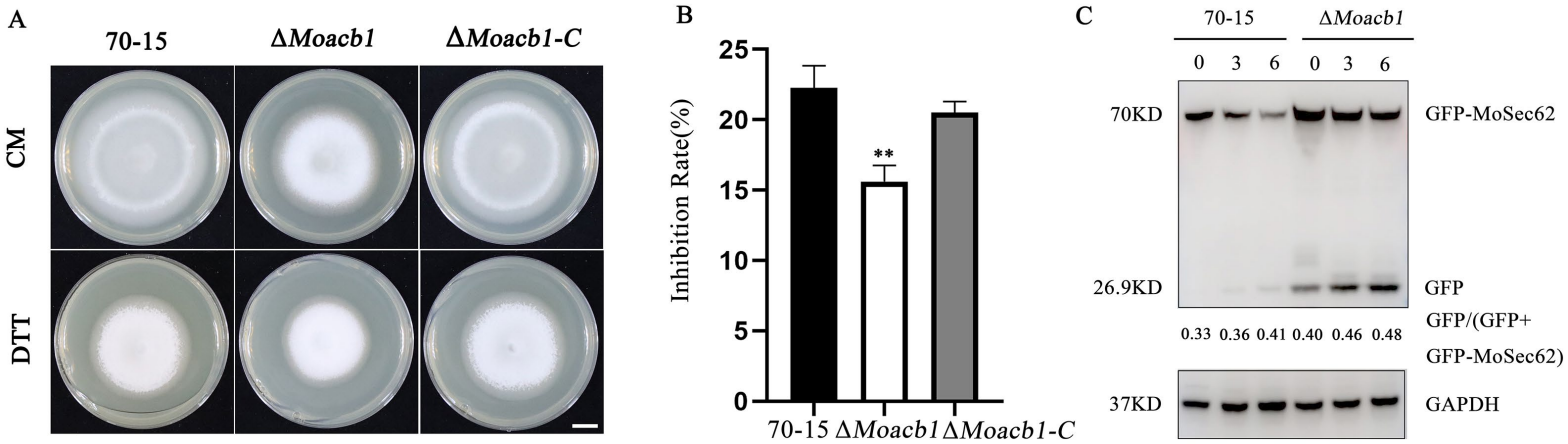
MoAcb1 participated in the analysis of ER autophagy. **(A)** The 70-15, ∆*Moacb1* and ∆*Moacb1-C* strains were inoculated on CM plates containing 2 mM DTT at 25°C for 8 days. Bar, 1 cm. **(B)** Relative inhibition rate of the 70-15, the ∆*Moacb1* and ∆*Moacb1-C* strains on CM medium containing 2 mM DTT, with the standard deviation indicated by error bars. *T*-test was used to test for significant differences. **p < 0.01. **(C)** Immunoblot analysis of MoSec62-GFP proteolysis in the 70-15 and ∆*Moacb1* mutant strains.

To test whether MoAcb1 regulates autophagy, we used Western blot to detect the content of full-length GFP-MoAtg8 and free GFP in the 70-15 and ∆*Moacb1* mutant (Nair et al., 2012). Under vegetative conditions, autophagy was weak, with few free GFP bands. Therefore, we tested the GFP-MoAtg8 activity induced under nitrogen starvation at 3 h and 6 h, respectively. The free GFP bands increased when the 70-15 and ∆*Moacb1* mutant were induced by nitrogen starvation medium (SD-N); however, there was no significant difference between the mutant and 70-15 (Supplementary Figure S3). Since this experiment found that MoAcb1 is mainly localized in the ER, We further detected whether ER-phagy was affected in the *ΔMoacb1* mutant. Strains were inoculated on CM containing 2 mM dithiothreitol (DTT, a chemical inducer of ER-phagy) and cultured for 8 days. The ∆*Moacb1* mutant strain showed greater resistance and grew better than the 70-15 and ∆*Moacb1-C* strain (Figures 7A,B). Next, we transferred GFP- MoSec62 (ER-phagy marker) into the 70-15 and ∆*Moacb1* mutant and found that 70-15 free GFP bands were not detected and that the GFP- MoSec62 band of the ∆*Moacb1* mutant was stronger than that of 70-15. After induction in liquid CM containing 5 mM DTT for 3 h and 6 h, respectively, the free GFP bands were increased in 70-15, and the GFP- MoSec62 band in the ∆*Moacb1* mutant remained numerous (Figure 7C). The results showed that the ER-phagy of the ∆*Moacb1* mutant was strengthened, and MoAcb1 played a negative role in regulating ER-phagy in *M. oryzae*.

## Discussion

Numerous studies have shown that acyl-CoA binding protein, which is frequently present in eukaryotic cells, has a high affinity for acyl-CoA. Acb protein participates in fatty acid oxidation in addition to transporting acyl-CoA (Sumper et al., 1969; Rasmussen et al., 1994). Fatty acid metabolism takes a variety of forms, which helps to show the variety of Acb protein gene functions and the flexibility of Acb protein genes in subcellular localization (Cohen Simonsen et al., 2003). Acb protein homologs were identified in animals, plants, fungi, protists and 11 eubacterial species. However, there are few studies on the function of the Acb protein in plant-pathogenic fungi. Whether the Acb1 protein plays a conserved function in blast fungus is still unknown. We knocked out *MoACB1* in wild-type 70-15 and found that MoAcb1 is involved in conidiation, appressorium development, and pathogenicity in *M. oryzae*.

Conidial germination in rice blast fungus produces appressoria. To achieve plant infection in the absence of exogenous nutrients, the appressorium uses autophagy pathway to breakdown the nutrients in conidia, continuously accumulates glycerol, and then produces enough turgor pressure to breach the host plant’s cuticle (Qu et al., 2020). In our study, the number of conidia produced by the ∆*Moacb1* mutant was significantly reduced, and the proportion of conidia with abnormal morphology was significantly increased. As shown in Figure 2C, the ∆*Moacb1-N2* could partly rescue the phenotype of conidia production, We suspect that it may be related to the fact that the ∆*Moacb1-N2* contains an Acb-binding domain. This indicated that ACB domain affected the conidiation of *M. oryzae*. In addition, the degradation rate of glycogen in the ∆*Moacb1* mutant was slowed, and the turgor pressure was also significantly reduced. The ∆*Moacb1* mutant almost completely lost its virulence. Acb protein partly regulates the β oxidation of fatty acids, the elongation of fatty acid chains, and lipid metabolism (Andersen et al., 1991). Therefore, we replaced the carbon sources in MM with different fatty acids, and we observed that the deletion of the *MoACB1* gene affected the utilization of different carbon sources by *M. oryzae*, indicating that the function of *MoACB1* of affecting the utilization of fatty acids was conserved in rice blast fungus.

Autophagy is an evolutionarily conserved intracellular degradation process in which some damaged proteins or organelles are encapsulated by double-membrane autophagy vesicles, then delivered to lysosomes or vacuoles to be degraded and cycled (Zhou et al., 2018; Zhu et al., 2021), thereby fulfilling the metabolic needs of the cells and the renewal of certain organelles. According to the different substrates, autophagy can be divided into macroautophagy and selective autophagy (Wang et al., 2022). In *Fusarium graminearum*, autophagy is required for normal vegetative growth, complete virulence, and toxin biosynthesis (Lv et al., 2017). In human and mouse cells, the Acb protein also regulates autophagy. Both intracellular depletion of the Acb protein and its addition to the extracellular environment inhibit autophagy (Knudsen et al., 1994). In our study, it was discovered that there was no discernible difference between the wild-type and the ∆*Moacb1* mutant in the rate of degradation of the autophagy-tagged protein GFP-MoAtg8, proving that MoAcb1 was not involved in macroautophagy. To maintain homeostasis in the ER, the endoplasmic reticulum associated protein degradation mechanism is initiated. In this study, We found that ∆*Moacb1* mutant was more resistant in CM plates containing 2 mM DTT, stimulating ER-phagy. As shown in Figure 7C, compared with 70-15, GFP-MoSec62 in ∆*Moacb1* mutant degrades more, We suspected that mutant require higher ER-phagy to maintain homeostasis.

In our study, we found that MoAcb1 is involved in ER-phagy, but the specific mechanism is unclear and needs to be further studied. MoAcb1 plays an important role in conidial growth, appressorium formation, hypertonic stress and pathogenicity of *M. oryzae*, providing a reference for subsequent exploration of other research aspects.

## Materials and methods

### Fungal strains and growth conditions

In this experiment, *M. oryzae* strain 70-15 was always used as the wild-type strain, and the mutant strain ∆*Moacb1* and the complemented strain ∆*Moacb1-C* were obtained by gene knockout. Unless stated, all strains were grown on CM plates and then grown in a 25°C incubator for 6–8 days (Yang et al., 2010). In the experiment to explore the ability to resist external environmental stress, the designated strains were inoculated on CM plates containing 0.5 M sodium chloride, 1 M sorbitol, 0.5 M potassium chloride and 1 M sucrose, cultivated at 25°C for 7 days and photographed. All assays in this study were performed in triplicate.

### Fluorescence localization

The conidia of MoAcb1-GFP strain were washed after 8 days of culture in CM. The conidia were stained with 1 μM ER-tracker (Invitrogen, America, E34250) dye solution at 28°C for 20 min and then fluorescence was observed with a laser confocal microscopy (LSM 880).

### Gene knockout and mutant complementation

Using a high-throughput gene knockout system (Lu et al., 2014), the *MoACB1* gene was knocked out, and then the copy of this gene was inserted into the deleted mutant to obtain the ∆*Moacb1-C* complement strain. To construct knockout vectors PKO3A, containing a suicide gene, *HSVtk*, we amplified upstream and downstream fragments of the target gene with primers and ligated the hygromycin B resistance gene (*HPH*) as a selectable marker (Supplementary Figure S4B; Liu et al., 2021). All these fragments were cloned using Phanta Max Ultra-Fidelity DNA Polymerase (Vazyme, China) and ligated into the *Hind*III/*Xba*I-cut linearized vector pKO3A (Lu et al., 2014). The recombination plasmids were transferred into *Agrobacterium tumefaciens* and the knockout was performed by ATMT (*Agrobacterium tumefaciens* mediated transformation) methods. The mutants were screened with CM plates containing the corresponding resistance and further verified by PCR. During the experiment, all PCR products and linearized vectors were verified by agarose gel electrophoresis (Supplementary Figure S4A) and then purified by a DNA gel extraction kit (Axygen, Hangzhou, China). In addition, qPCR confirmed only one copy of the *HPH* gene in *MoABC1* deletion mutant (Supplementary Figure S4D) and the *MoACB1* gene could not detected in *MoACB1* deletion mutant (Supplementary Figure S4C). Relative expression was calculated according to 2^−ΔΔCT^. Actin gene was used as the reference gene of qPCR. For the complementation assay, the target gene *MoACB1* was amplified with the upstream promoter, fused with the PKD5-GFP vector containing sulfonylurea resistance gene (SUR), and transferred into the Δ*Moacb1* mutant to obtain the complement strain Δ*Moacb1-C*. Complement strains were identified by phenometric analysis, fluorescence observation and qPCR. The PCR primers used in this study are listed in Table 1.

**Table 1:** Primers in this study.

Name Sequence (5′ - 3′)	
*Primers used for gene knockout*	
HPH-F	TAGTGGAGGTCAACAATGAATG
HPH-R	CATCTACTCTATTCCTTTGCCC
ACB1up-F	AGGCTAACTGACACTCTAGAGAGATGCCGGTTTAATTCCCC
ACB1up-R	TGTTGACCTCCACTAAAGCTGGCAGGTGTTCGTGCTC
ACB1dn-F	GGAATAGAGTAGATGAAACCAAAATATCGCGGAG
ACB1dn-R	CGACGGCCAGTGCCAAGCTTTGAAGTGGTGACACAGAT
ACB1inner-F	AAGGGATCAATCTCGTCTTCAG
ACB1inner-R	TTTTTGCCAGGCGTTCTTCTTG
ACB1upyz-F	TCTCAAGAGGCAAAGATGGC
ACB1upyz-R	GTCGGAGACGCTGTCGAACTT
*Primers used for complement assay*	
ACB-N1-GFP-F	ATCACAATGGCCGGATCCATGTCATCCGTCACTTTGGTA
ACB-N1-GFP-R	CTTGCTCACCATCCCGGGTGCTGGAGCCATGTTTGATGA
ACB-N2-GFP-F	ATCACAATGGCCGGATCCCAGTCCGAGGCTTTCCAGAAG
ACB-N2-GFP-R	CTTGCTCACCATCCCGGGCAAAGCCATAGGCGGTCTTCA
*Primers used in fluorescent observation*	
ACB1-GFPF	ACAATCACTAGTGAATTCTGACATGTGGGTGGCCTCCAA
ACB1-GFPR	CATCCCGGGGATGGATCCAGCGCCAACGGCCTCAGGGAC
*Primers used for qPCR*	
qRT-HPH-F	ATGTCCTGCGGGTAAATAGC
qRT-HPH-R	GATGCAATAGGTCAGGCTCTC
qRT-Actin-F	ACAATGGTTCGGGTATGTGC
qRT-Actin-R	CGACAATGGACGGGAAGAC
qRT-ACB1-F	TGTCATCCGTCACTTTGGTAC
qRT-ACB1-R	GACTGAAGACGAGATTGATCCC

### Phenotypic characterization

For vegetative growth and conidiation experiments, the strains were grown in CM. Eight days after inoculation, colony diameter and conidiation were determined. For the conidial germination test, the conidia were washed out and diluted to 5 × 10^4^ cells/ml, and the suspensions were induced on the hydrophobic membrane for 4 h, 6 h, 8 h, and 24 h (Kong et al., 2012).

For pathogenicity assays, isolated barley leaves were inoculated with 70-15, ∆*Moacb1* and ∆*Moacb1-C* mycelial plugs and conidial suspensions (5 × 10^4^ cells/ml). The cells were cultured at 25°C for 4 days, and the diseased spots were observed and photographed (Kim et al., 2005). Moreover, we performed live-cell imaging using isolated barley leaves and leaf sheaths to observe how invasive hyphae developed within the host cell. Conidia suspension was inoculated in leaves and leaf sheaths and cultured in a wet box to observe and recorded the infection at different time periods (Zhou et al., 2009; Fernandez and Wilson, 2014). As previously mentioned, we measured the turgor pressure of appressoria using glycerol solutions of 0.5 M, 1.0 M, 1.5 M, and 2.0 M. Glycogen was stained with KI/I_2_ solution to detect its breakdown. Each of the aforementioned experiments was carried out three times.

### Nutrient replacement and growth stress test

To test whether there is a difference in the utilization of different nutrients by mutants, we replaced the carbon and nitrogen sources in MM, respectively. In the carbon source utilization experiment, 1% glucose in the medium is replaced by sodium acetate, olive oil, palmitic acid, ferulic acid, and tetradecanoic acid, respectively. In the nitrogen source utilization experiment, 10 mM of NaNO_3_ is replaced by NaNO_2_, (NH_4_)_2_SO_4_, NH_4_NO_3_, Gln and His, respectively. The strain is incubated on plates for 8 days and the diameter of each sample is measured to calculate the growth rate. The procedure was repeated 3 times for each strain.

To test the response of mutants to hyperosmotic stress, CM containing 0.5 M potassium chloride, 1 M sucrose, 0.5 M sodium chloride, and 1 M sorbitol was prepared. To test the susceptibility of mutants to DNA damage reagents, CM containing 20 mM HU and 0.02%MMS was prepared. The strains were incubated on plates for 8 days and the diameter of each sample was measured to calculate the inhibition rate. The procedure was repeated 3 times for each strain.

### Autophagy induction and western blot analysis

To detect autophagy flux, we transferred GFP-MoAtg8 into 70-15 and ∆*Moacb1* mutant. The transferred strains were cultured in liquid CM at 25°C and 150 rpm for 48 h, followed by induction in SD-N medium for 3 h and 6 h to induce autophagy. To induce ER-phagy, GFP-MoSec62 was transferred into 70-15 and ∆*Moacb1* mutant. The mycelia of strain were transferred into SD-N with 5 mM DTT, the culture conditions are the same as above. For autophagy and ER-phagy, the free GFP and fusion bands were detected by GFP antibody (Huabiao, Hangzhou, China) with 12% SDS-PAGE. The protein GAPDH was used as a loading control.

## Data availability statement

The original contributions presented in the study are included in the article/Supplementary material, further inquiries can be directed to the corresponding author.

## Author contributions

LL, X-MZ, and J-DB designed all the experiments. L-HZ and HL participated in data statistical analysis. NC wrote the first draft. LL, X-MZ, and F-CL provided fund support. All authors contributed to the article and approved the submitted version.

## Funding

This work is supported by grants (32200162 to LL, 31970140 to F-CL, 32100159 to X-MZ) from the National Natural Science Foundation of China.

## Conflict of interest

The authors declare that the research was conducted in the absence of any commercial or financial relationships that could be construed as a potential conflict of interest.

## Publisher’s note

All claims expressed in this article are solely those of the authors and do not necessarily represent those of their affiliated organizations, or those of the publisher, the editors and the reviewers. Any product that may be evaluated in this article, or claim that may be made by its manufacturer, is not guaranteed or endorsed by the publisher.

## Footnotes

^1^http://fungi.ensembl.org/Magnaporthe_oryzae/Info/Index
