# Supplementary figures and images for: Acyl-coenzyme A binding protein MoAcb1 regulates conidiation and pathogenicity in Magnaporthe oryzae
Source: Front Microbiol. 2023 Apr 28;14:1179536. doi: 10.3389/fmicb.2023.1179536 (PMC10175604; doi:10.3389/fmicb.2023.1179536)

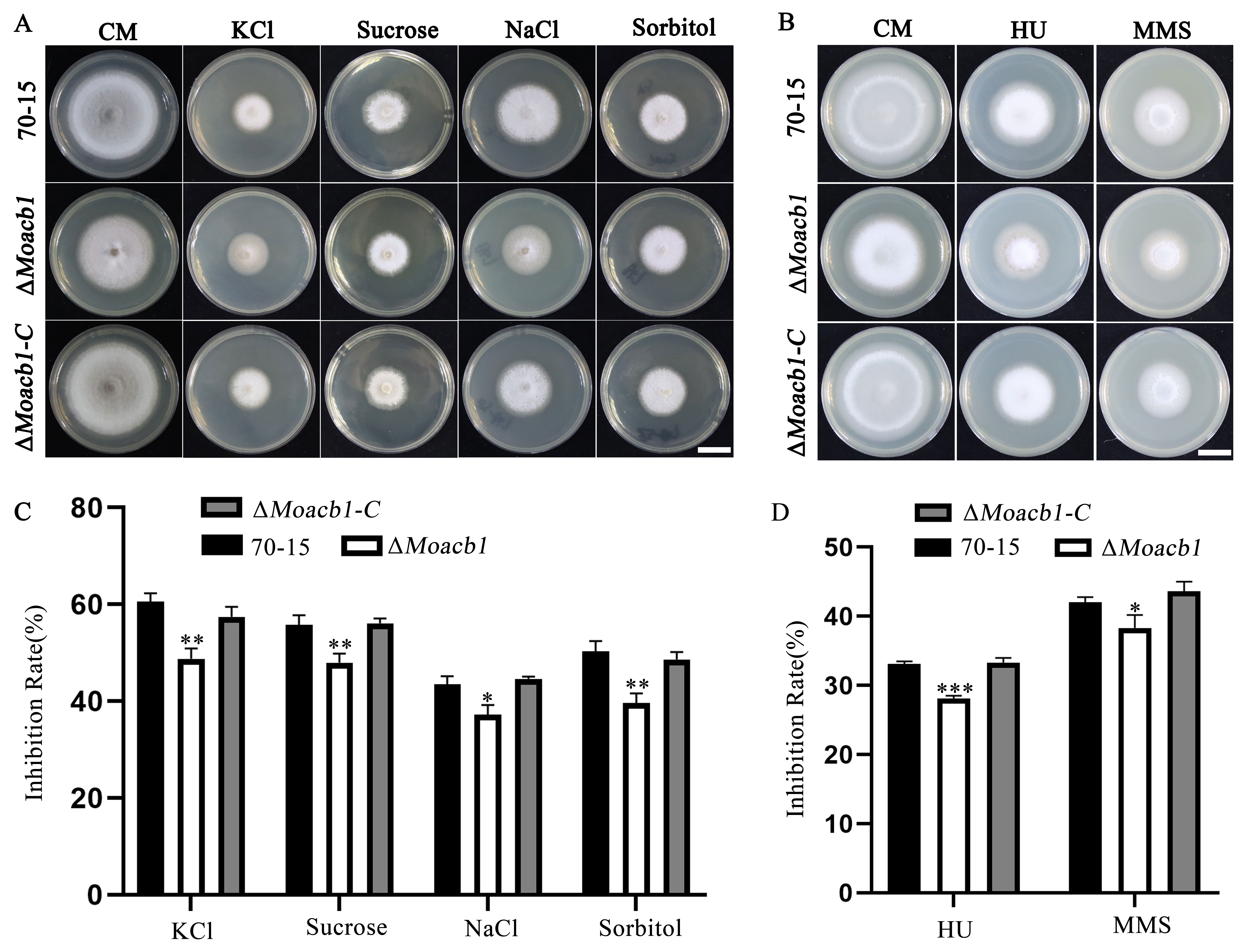

Supplement: Supplementary file 2 [file image_2.jpeg]

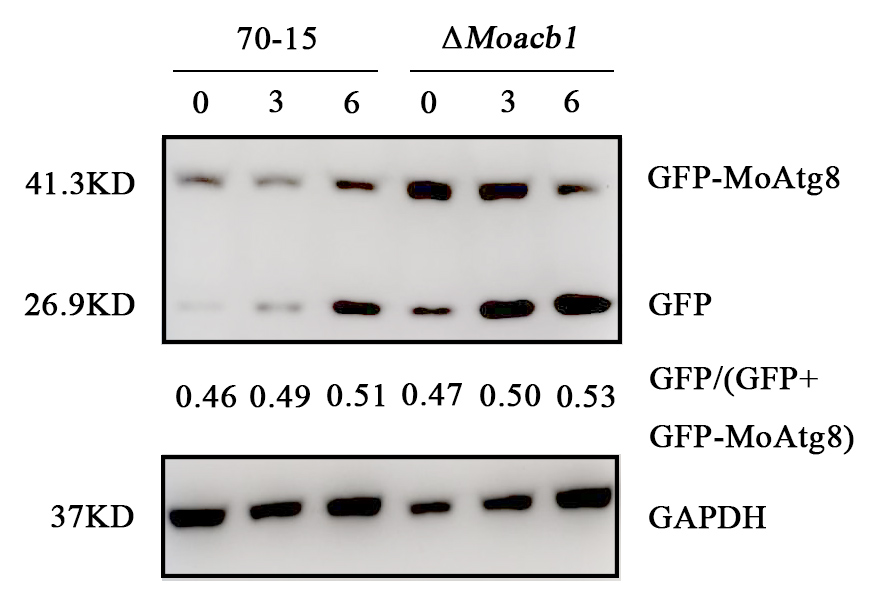

Supplement: Supplementary file 3 [file image_3.jpeg]

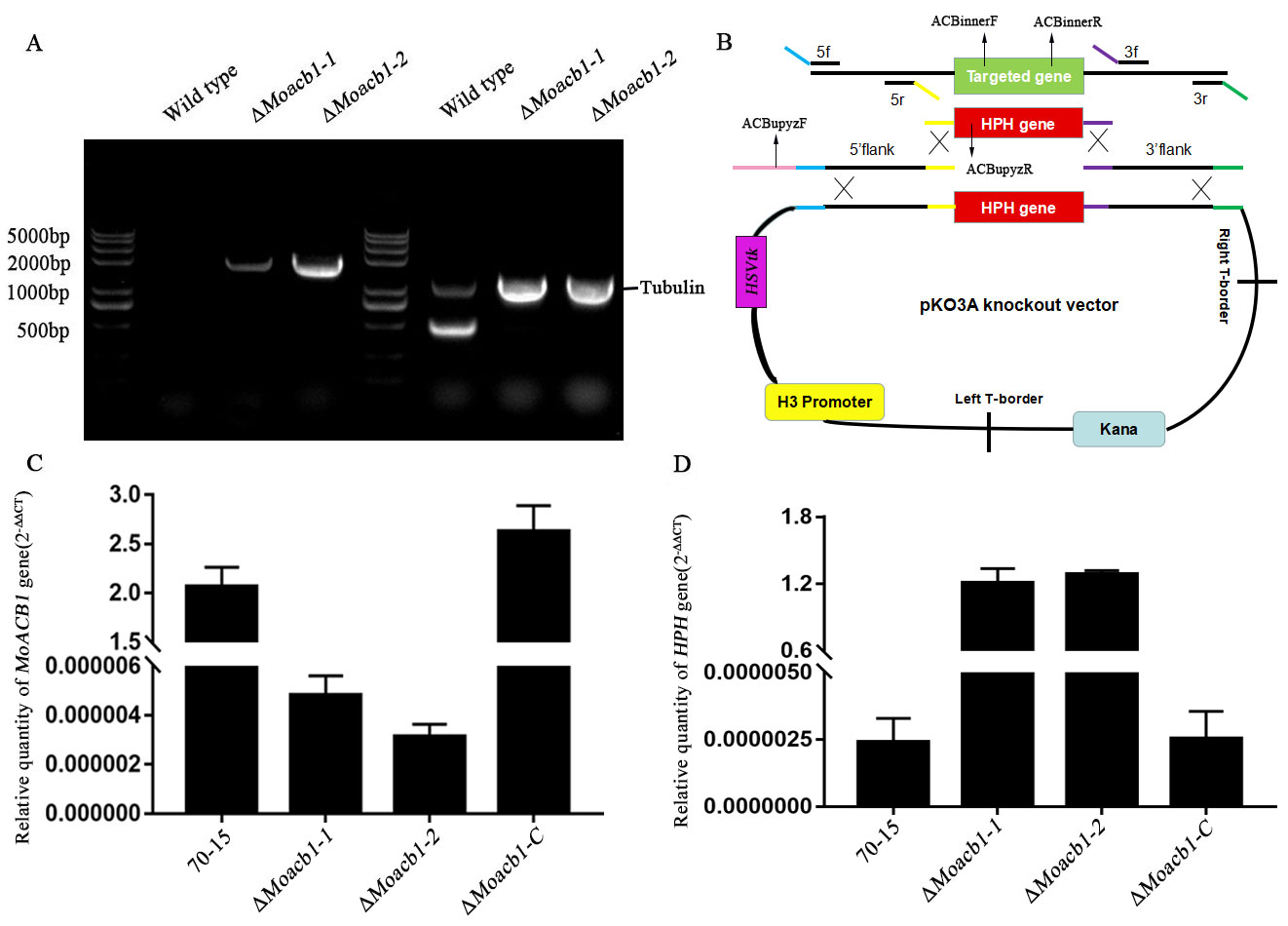

Supplement: Supplementary file 4 [file image_4.jpeg]
