# Supplementary material for: Acyl-coenzyme A binding protein MoAcb1 regulates conidiation and pathogenicity in Magnaporthe oryzae
Source: Front Microbiol. 2023 Apr 28;14:1179536. doi: 10.3389/fmicb.2023.1179536 (PMC10175604; doi:10.3389/fmicb.2023.1179536)
Supplement: Supplementary file 5 [file data_sheet_1.docx]

**Supplementary material**

**Figure S1.Utilization of different carbon and nitrogen sources by *M. oryzae*.**

A. The 70-15, ∆*Moacb1* and ∆*Moacb1-C* strains were inoculated on MM medium containing 50 mM sodium acetate, olive oil, palmitic acid, ferulic acid, and tetradecanoic acid. Bar, 1 cm.

B. The relative inhibition rates of the 70-15, ∆*Moacb1* and ∆*Moacb1-C* strains were calculated under MM medium conditions containing different carbon sources. Each experiment was performed in triplicate, with the standard deviation indicated by error bars. T-test was used to test for significant differences. *P < 0.05, **P < 0.01.

C. The 70-15, ∆*Moacb1* and ∆*Moacb1-C* strains were inoculated on MM medium containing NaNO_2_, (NH_4_)_2_SO_4_, NH_4_NO_3_, glutamine, and histidine at 25°C for 8 days. Bar, 1 cm.

D. The relative inhibition rates of the 70-15, ∆*Moacb1* and ∆*Moacb1-C* strains were calculated under MM medium conditions containing different nitrogen sources. Each experiment was performed in triplicate, with the standard deviation indicated by error bars. T-test was used to test for significant differences. *P < 0.05, **P < 0.01, ***P < 0.001.

**Figure S2. Deletions of *MoACB1* increase the resistance of strains to hypertonic stress and DNA replication stress**

A. 70-15, ∆*Moacb1* and ∆*Moacb1-C* strains were inoculated on CM plates containing 0.5 M potassium chloride, 1 M sucrose, 0.5 M sodium chloride, and 1 M sorbitol. Bar, 1 cm.

B. 70-15, ∆*Moacb1* and ∆*Moacb1-C* strains were inoculated on CM plates containing 20 mM HU and 0.02% MMS. Bar, 1 cm.

C. 70-15, ∆*Moacb1* and ∆*Moacb1-C* strains showed the relative inhibition rate on CM medium containing 0.5 M potassium chloride, 1 M sucrose, 0.5 M sodium chloride, and 1 M sorbitol, with standard deviation indicated by error bars. T-test was used to test for significant differences. *P < 0.05,**P < 0.01.

D. 70-15, ∆*Moacb1* and ∆*Moacb1-C* strains showed the relative inhibition rates on CM medium containing 20 mM HU and 0.02% MMS, with standard deviation indicated by error bars. T-test was used to test for significant differences. *P < 0.05, ***P < 0.001.

**Figure S3. Western blot analysis**

Immunoblot analysis of the degradation of GFP-MoAtg8 was detected in 70-15 and ∆*Moacb1* mutant strains under nitrogen starvation for 0, 3, and 6 h by GFP antibodies. GAPDH was used as an internal reference for protein standardization by Western blot. The degradation rates were calculated by the formula: GFP/(GFP+GFP-MoAtg8).

**Figure S4. Identification of the knockout genes and verification of the copy number**

1. The targeted gene in the transformant was screened by PCR using the tubulin gene as a positive control. A characteristic short fragment was amplified from wild-type 70-15 and randomly inserted transformants, indicating that the target gene was present. Transformants were then screened again by the PCR to amplify a long 1.5-2.5 kb fragment from the knockout mutants, whereas the wild-type 70-15 and random insertion transformants could not amplify.

B. The 5’ and 3’ flanking fragments of the targeted genes were separately amplified from genomic DNA with the primers 5f/5r and 3f/3r. The 5r and 3f primers are homologous to the HPH cassette, whereas the 5f and 3r primers are homologous to the vector. The three amplified fragments and the *Xba*I/*Hind*III-linearization vector pKO3A were ligated together with ligase. Homologous recombination produced a circular knockout vector, which was subsequently used to transform into *A. tumefaciens*.

C. The relative expression level of *MoACB1* was verified by qPCR in the 70-15, ∆*Moacb1* mutant and ∆*Moacb1-C*. Actin gene was used as control gene. Relative expression was calculated based on 2^-∆∆CT^.

D. The relative expression level expression of resistance gene *HPH* was verified by qPCR in the 70-15, ∆*Moacb1* mutant and ∆*Moacb1-C*. Actin gene was used as control gene. Relative expression was calculated based on 2^-∆∆CT^.
